# Comparing Fear of COVID-19 and Preventive COVID-19 Infection Behaviors Between Iranian and Taiwanese Older People: Early Reaction May Be a Key

**DOI:** 10.3389/fpubh.2021.740333

**Published:** 2021-09-23

**Authors:** Amir H. Pakpour, Chieh-hsiu Liu, Wen-Li Hou, Yu-Pin Chen, Yueh-Ping Li, Yi-Jie Kuo, Chung-Ying Lin, Damian Scarf

**Affiliations:** ^1^Social Determinants of Health Research Center, Research Institute for Prevention of Non-communicable Diseases, Qazvin University of Medical Sciences, Qazvin, Iran; ^2^Department of Nursing, School of Health and Welfare, Jönköping University, Jönköping, Sweden; ^3^National Cheng Kung University Hospital, College of Medicine, National Cheng Kung University, Tainan, Taiwan; ^4^Department of Nursing, Kaohsiung Medical University, Kaohsiung, Taiwan; ^5^Department of Medical Research, Kaohsiung Medical University Hospital, Kaohsiung, Taiwan; ^6^Department of Orthopedic Surgery, Wan Fang Hospital, Taipei Medical University, Taipei, Taiwan; ^7^Department of Orthopedic Surgery, School of Medicine, College of Medicine, Taipei Medical University, Taipei, Taiwan; ^8^Department of Nursing, National Tainan Junior College of Nursing, Tainan, Taiwan; ^9^Institute of Allied Health Sciences, College of Medicine, National Cheng Kung University, Tainan, Taiwan; ^10^Department of Public Health, National Cheng Kung University Hospital, College of Medicine, National Cheng Kung University, Tainan, Taiwan; ^11^Department of Occupational Therapy, College of Medicine, National Cheng Kung University, Tainan, Taiwan; ^12^Department of Psychology, University of Otago, Dunedin, New Zealand

**Keywords:** COVID-19, elder, infection preventive behavior, Iran, Taiwan

## Abstract

This study assessed fear of the novel coronavirus-2019 (COVID-19), preventive COVID-19 infection behaviors, and the association between fear of COVID-19 and preventive COVID-19 infection behaviors among older people in Iran and Taiwan. Older people aged over 60 years (*n* = 144 for Iranians and 139 for Taiwanese) completed the Fear of COVID-19 Scale (FCV-19S) and two items on preventive COVID-19 infection behaviors (i.e., hand washing and mouth covering when sneezing). Iranian older people had a significantly higher level of fear of COVID-19 than did Taiwanese older people. Moreover, Iranian older people had significantly lower frequencies of preventive COVID-19 infection behaviors than did Taiwanese older people. Different timings in implementing COVID-19 infection control policies in Iran and Taiwan may explain why Iranian older people had greater fear of COVID-19 and lower preventive COVID-19 infection behaviors than did Taiwanese older people.

## Introduction

Depression, anxiety, and stress are relatively common among older adults (1, 2). Indeed, nearly 10% of older adults suffer from depressive and anxiety symptoms (3). Beyond their direct impact, mood disorders make a significant contribution to a range of poor health outcomes (4, 5). Therefore, understanding the factors related to mood problems among older adults is an important area of research. To date, several factors have been reported, including sleeping quality and underling disorders such as cerebellar degenerative disease (6, 7). Beyond the common factors reported in the literature, the novel coronavirus-2019 (COVID-19) has the potential to significantly impact both the mental and physical health of older adults. Moreover, the impacts of COVID-19 on individuals' stress and mental health have been frequently reported worldwide (8–12).

COVID-19 has a rapid transmission rate and has quickly spread worldwide, with nearly 5.6 million confirmed cases and more than 350,000 deaths at the time of writing (29 May 2020). Therefore, the World Health Organization (WHO) has announced that the COVID-19 infection is a global pandemic (13). The impact of COVID-19 may be especially severe for older adults, with recent evidence demonstrating that older people are especially vulnerable to COVID-19 infection (14). More specifically, the mortality rates of COVID-19 infection for people aged over 70 are 12.8% in Italy and 8.0% in China (14). Further, the mortality of COVID-19 infection is highly associated with chronic diseases, especially those that tend to be more common among older adults, such as cardiovascular disease, dementia, Parkinson's disease, and cancer (14–17). Given their increased risk, older adults may be more fearful of COVID-19 which could, in turn, contribute to the increased incidence of stress and anxiety associated with the COVID-19 pandemic (18, 19).

Recently, Ahorsu et al. developed the Fear of COVID-19 Scale (FCV-19S) (20). The FCV-19S was completed by an Iranian sample and revealed that there was a very high level of fear in the peak of COVID-19 outbreak; that is, March 2020 (21). An appropriate amount of fear motivates an individual to perform appropriate preventive behaviors (22). Indeed, several behavioral theories (e.g., the Protection Motivation Theory, the Health Believe Model, and the Fear Drive Model) have proposed that fear may lead people to adhere to healthy behaviors and decrease unhealthy behaviors, such as increasing exercise while also quitting smoking (23, 24). At the same time, high levels of fear may negatively impact health (25, 26). Therefore, finding the right balance with respect to the fear of COVID-19 may be critical to encouraging adherence to behaviors that aim to prevent the spread of COVID-19.

The present study investigated fear of COVID-19 and preventive COVID-19 infection behaviors among older people from two different countries (Iran and Taiwan). By comparing these two countries, the present study aims to shed some light on the effectiveness of the different public health approaches applied in these two countries in response to the COVID-19 outbreak. More specifically, both Iranian and Taiwanese governments used universal policies (e.g., boarder control, encouragement of preventive COVID-19 infection behaviors, and instant reporting of COVID-19 information through different social media platforms) to control the spread of COVID-19. The Taiwanese government, however, had a much quicker response to the COVID-19 pandemic than the Iranian government. For example, the Taiwanese government implemented infection control policies in late January, while the Iranian government only implemented similar policies in late February. Moreover, during the initial COVID-19 outbreak period, the Iranian government canceled sporting events and closed public places, with a lockdown conducted between 28 March and 9 April 2020. In contrast, the Taiwanese government did not cancel large events and did not close public places, instead using strict regulations during events and in public places (e.g., requiring face masks, enforcing physical distancing).

Regarding the COVID-19 infection development in Iran, Qom had the earliest confirmed cases of COVID-19 (on 19 February 2020). Following this, an additional 18 cases, four of whom died, were reported 2 days later (on 21 February). The increase of COVID-19 infection cases grew dramatically between February and March, with 16,169 confirmed cases and 988 deaths by 17 March. In order to control the COVID-19 outbreak, the Iranian government disseminated guidelines and related information via TV, SMS, and the internet. Moreover, the government set up COVID-19 hotlines to answer COVID-19 queries from the general population (27). By 29 May, 2020, there were 143,849 confirmed cases and 7,627 deaths.

Regarding the COVID-19 situation in Taiwan, the earliest confirmed case of COVID-19 occurred on 21 January 2020 and the first death was reported nearly 1 month later (on 16 February). The increase of COVID-19 infection cases was generally well-controlled from January to May, with 441 confirmed cases and 7 deaths by 28 May. Moreover, only 91 cases were infected in the community, with the remaining 350 infected abroad. Similar to the Iranian government, the Taiwanese government disseminated guidelines and related information though standard channels (i.e., TV, SMS, and the internet) and set up COVID-19 hotlines to answer COVID-19 queries from the general population. Furthermore, the Taiwanese government paid special attention to the COVID-19 infection development in the early stages, with border control, quarantine, and isolation all being implemented since the first infection was confirmed. The government also responded to all the potential risks of COVID-19 transmission, such as cruise ships coming to Taiwan (28, 29). For example, the Diamond Princess cruise ship, which reported COVID-19 outbreak at the Yokohama on 5 February 2020, had been docked at Keelung harbor in Taiwan on 31 January 2020. With the aforementioned information reported by the media, a temporary public panic concerning the risk of community spread was triggered (28, 30). Thus, the government implemented additional precautionary measures, including comprehensive contact tracing and a mitigation plan to minimize COVID-19 infection spread (28).

Table 1 further summarized the COVID-19 situations between Iran and Taiwan. In brief, the Taiwan government seemed to respond more quickly to the COVID-19 outbreak when compared to the Iran government. Therefore, the infection status of COVID-19 was different between the two countries, and this may subsequently lead to differences in preventive behaviors and the degree of fear between the two countries' older people. In this regard, the present study hypothesized that (i) Iranian older people will report higher levels of fear of COVID-19 than Taiwanese older people; (ii) the association between fear of COVID-19 and preventive COVID-19 infection behaviors will be stronger in Iranian older people than in Taiwanese older people.

**Table 1:** COVID-19 situations and present study's recruitment information between Iran and Taiwan.

**Comparisons**	**Iran**	**Taiwan**
Data collection period in the present study	Entire April 2020	Late April to early May 2020
Recruitment method in the present study	Online from community population	In-person from patient population
The earliest confirmed cases of COVID-19 infection	19 February 2020 (in Qom)	21 January 2020 (in Taoyuan airport)
The earliest death(s)	21 February 2020 (4 deaths)	16 February 2020 (1 death)
Growth of COVID-19 infection cases	Between February and March (16169 confirmed cases with 988 deaths) Between February and May (143849 confirmed cases with 7627 deaths)	Between January and May (441 confirmed cases with 7 deaths)
Government action	1. Disseminating guidelines and related information via TV, SMS, and online resources 2. Setting up COVID-19 hotlines to answer COVID-19 queries	1. Disseminating guidelines and related information via TV, SMS, and online resources 2. Setting up COVID-19 hotlines to answer COVID-19 queries 3. Paying special attention to the COVID-19 infection development in the early stages. Border control, quarantine, and isolation all being implemented since the first infection was confirmed.

## Methods

Taipei Medical University's ethical committee approved the study with registered numbers of TMU-JIRB N202005044. Also, ethics committee of Qazvin University of Medical Sciences approved the study with registered number of IR.QUMS.REC.1398.375.

### Participants and Recruitment Procedure

For recruitment of Iranian older adults, online social media platforms, including telegram, Instagram, and WhatsApp, were used. The three online platforms are the most popular social media platforms in Iran, and the link that described study aims and descriptions together with questionnaires was posted on these platforms. The Iranian data were collected throughout April 2020. For recruitment of Taiwanese older people, the target population were older adult patients who visited and consulted a physician from one medical center in Taipei, Taiwan. During their visits, several research assistants explained the study aims and descriptions to them. Then, the research assistants let the participants who agreed to participate in the study sign a written informed consent form before interviewing them using the survey questionnaires. All the interviews were done face-to-face and were administered in a private room. Similar to the timeline in Iran, the Taiwanese data were collected from late April to early May 2020. Different methods in data collection were applied because during the survey period, Iran had severe COVID-19 outbreak and it was unable to approach the participants in person. However, Taiwan was in mild severity of COVID-19 outbreak and we believed that completing the survey using face-to-face method can better control the data quality.

The inclusion criteria of the present study's participants were (i) aged 60 years and above; (ii) voluntarily agreeing to participate in the study; and (iii) the ability to understand the survey questions. There were no other exclusion criteria for the participants.

### Measures

#### Fear of COVID-19

Fear of COVID-19 was measured using a well-established instrument [i.e., Fear of COVID-19 Scale; FCV-19S; (20)]. The FCV-19S includes seven items that assess an individual's fear toward COVID-19 with a five-point Likert scale (1 = strongly disagree; 5 = strongly agree). A higher score in the FCV-19S indicates greater fear of COVID-19. Moreover, the FCV-19S has promising psychometric properties in different language versions, including Persian (20), Bangla (31), Russian (32), Turkish (33), Italian (34), Arabic (35), and Hebrew (36). For example, the unidimensional structure of the FCV-19S has been verified using both confirmatory factor analysis and exploratory factor analysis (20, 31). The internal consistency of the FCV-19S in the present sample was satisfactory: α = 0.79 for Taiwanese older people and 0.91 for Iranian older people.

#### Preventive COVID-19 Infection Behaviors

Two preventive COVID-19 infection behaviors were designed according to the suggestions made by the World Health Organization (WHO) to fight COVID-19 (37). The two behaviors are hand washing and mouth covering when sneezing, and they were measured using a five-point Likert scale (1 = almost never; 5 = almost always). Thus, a higher score indicates higher frequencies in performing these preventive behaviors. Although the WHO proposed other three behaviors of wearing a mask, physical distancing, and avoid touching eyes nose mouths, the present study did not assess the three behaviors because of the following reason. The use of mask for prevention was still under debate (38) during our data collection period. Therefore, we did not assess this behavior. Regarding physical distancing and avoid touching eyes nose mouth, we considered that the two behaviors are very likely to be misreported. Specifically, one usually moves close to another person and was not aware of this action when they are talking. Similarly, one usually touches his/her eyes, nose, or mouth unawareness. Therefore, the present study mainly focused on the behaviors of hand washing and mouth covering when sneezing.

#### Background Information

A background information sheet was used to measure the participants' demographic and clinical characteristics. More specifically, the demographic information included the participants' age, gender, living area (urban or not), and educational level. The clinical characteristics included the following chronic diseases: diabetes mellitus, hypertension, heart disease, renal disease, and cancer.

### Data Analysis

Descriptive statistics, including mean with standard deviation (SD) and frequency with percentage, were firstly carried out to understand the demographics and clinical characteristics of the participants. Moreover, the participants' fear of COVID-19 and preventive COVID-19 infection behaviors were presented using descriptive statistics. Then, independent *t*-tests and χ^2^-tests were applied to examine whether there were significant differences between Iranian and Taiwanese older people. Lastly, four regression models were constructed to examine the factors that explain fear of COVID-19 and abiding by preventive COVID-19 infection behaviors. More specifically, the first regression model used fear of COVID-19 as the dependent variable; demographics (age, gender, education, and living area), clinical characteristics (having a diabetes mellitus, hypertension, heart disease, renal disease, and cancer), and group (Iranian or Taiwanese older people) as independent variables. The second to the fourth regression models used hand washing, mouth covering when sneezing, and total behavior (i.e., adding both hand washing and mouth covering behaviors) as the dependent variables, respectively. Moreover, the independent variables were the same as those used in the first regression model, with the addition of the FCV-19S. All the statistical analyses were performed using the IBM SPSS 24.0 (IBM Corp., Armonk, NY).

## Results

Participants' demographic and clinical characteristics are presented in Table 2, which shows that the Iranian sample (*n* = 144; mean = 65.59; *SD* = 6.65) was significantly younger than the Taiwanese sample (*n* = 139; mean = 71.73; *SD* = 7.90; *p* < 0.001). Moreover, the Taiwanese sample had significantly more females (69.8 vs. 29.2%; *p* < 0.001), were better education (2.9% illiterate vs. 24.3% illiterate; *p* < 0.001), and had more participants living in urban areas (91.4 vs. 50.7%; *p* < 0.001). Regarding chronic diseases, no significant differences were found in the percentages of having hypertension and cancer between the two groups. However, Taiwanese sample as compared with Iranian sample had a significantly lower prevalence of diabetes mellitus, heart disease, and rental disease (*p*s < 0.001). A significantly higher level of fear of COVID-19 was observed in Iranian older people (mean = 3.36; *SD* = 1.04) when compared to Taiwanese older people (mean = 1.80; *SD* = 0.80; *p* < 0.001). Interestingly, a significantly lower frequencies of preventive COVID-19 infection behaviors were observed in Iranian older people (mean = 4.06–4.15; *SD* = 0.89–1.03) when compared to Taiwanese older people (mean = 4.78–4.86; *SD* = 0.54–0.68; *p*s < 0.001).

**Table 2:** Comparing participants' characteristics, fear of COVID-19, and behaviors between Iranian and Taiwanese older people.

	**Mean (SD) or** ***n*** **(%)**		***t* or χ^**2**^ (*p*-value)**
	**Iranian (*N* = 144)**	**Taiwanese (*N* = 139)**	
Age (year)	65.59 (6.65)	71.73 (7.90)	7.07 (<0.001)
Gender (female)	42 (29.2)	97 (69.8)	46.69 (<0.001)
Education			48.03 (<0.001)
Illiterate	35 (24.3)	4 (2.9)	
Primary school	15 (10.4)	31 (22.3)	
Secondary school	15 (10.4)	45 (32.4)	
Diploma or above	79 (54.9)	59 (42.4)	
Living area (urban)	73 (50.7)	127 (91.4)	56.45 (<0.001)
**Chronic disease (no)**			
Diabetes mellitus	81 (56.3)	115 (82.7)	23.30 (<0.001)
Hypertension	79 (54.9)	88 (63.3)	2.09 (0.15)
Heart disease	106 (73.6)	122 (87.8)	9.06 (0.003)
Renal disease	126 (87.5)	134 (96.4)	7.51 (0.01)
Cancer	124 (86.1)	125 (89.9)	0.39 (0.53)
Fear of COVID-19	3.36 (1.04)	1.80 (0.80)	14.22 (<0.001)
Hand washing	4.15 (0.96)	4.78 (0.68)	6.43 (<0.001)
Mouth covering	4.06 (1.03)	4.86 (0.57)	8.17 (<0.001)
Total behavior	4.11 (0.89)	4.82 (0.54)	8.24 (<0.001)

*Mouth covering indicates covering mouth when sneezing*.

*Total behavior includes both hand washing and mouth covering when sneezing*.

The regression models supported this finding that Iranian older adults had greater fear of COVID-19 than Taiwanese older adults (standardized coefficient [β] = 0.60; adjusted odds ratio [AOR] = 1.82; *p* < 0.001) but performed fewer preventive COVID-19 infection behaviors (β = −0.42 to −0.58; AOR = 0.66–0.56; *p*s < 0.001) when demographics and clinical characteristics were controlled (Table 3). Fear of COVID-19 was another significant predictor in explaining older people's preventive COVID-19 infection behaviors: greater fear was associated with more preventative COVID-19 infection behaviors (β = 0.27, AOR = 1.31, and *p* < 0.001 for hand washing; β = 0.14, AOR = 1.15, and *p* = 0.01 for mouth covering when sneezing; β = 0.26, AOR = 1.30, and *p* < 0.001 for total behavior).

**Table 3:** Regression models in explaining fear of COVID-19 and preventive COVID-19 behaviors.

	**B (SE)/β** **(*****p*****-value)**			
	**Fear of COVID-19**	**Hand washing**	**Mouth covering**	**Total behavior**
Age	0.02 (0.01)/0.12 (0.02)	0.01 (0.01)/0.05 (0.45)	−0.01 (0.01)/−0.09 (0.13)	−0.003 (0.01)/−0.03 (0.67)
Gender (ref: female)	0.10 (0.13)/0.04 (0.42)	−0.36 (0.11)/−0.20 (0.001)	−0.27 (0.11)/−0.15 (0.02)	−0.32 (0.10)/−0.19 (0.002)
Education^a^	0.03 (0.12)/0.01 (0.78)	−0.02 (0.11)/−0.01 (0.85)	0.04 (0.11)/0.02 (0.72)	0.01 (0.09)/0.01 (0.93)
Living area (Ref: urban)	0.25 (0.13)/0.10 (0.06)	−0.11 (0.12)/−0.06 (0.38)	0.13 (0.12)/0.07 (0.27)	0.01 (0.11)/0.01 (0.90)
**Chronic disease (Ref: no)**				
Diabetes mellitus	−0.03 (0.13)/−0.01 (0.81)	0.01 (0.11)/0.002 (0.97)	−0.12 (0.11)/−0.06 (0.29)	−0.06 (0.10)/−0.03 (0.56)
Hypertension	−0.01 (0.11)/−0.004 (0.92)	0.08 (0.10)/0.04 (0.46)	0.29 (0.10)/0.16 (0.005)	0.18 (0.09)/0.11 (0.04)
Heart disease	0.29 (0.14)/0.10 (0.04)	0.15 (0.13)/0.07 (0.24)	0.17 (0.13)/0.07 (0.19)	0.16 (0.11)/0.08 (0.16)
Renal disease	0.37 (0.20)/0.08 (0.07)	0.03 (0.18)/0.01 (0.88)	0.16 (0.19)/0.05 (0.39)	0.09 (0.16)/0.03 (0.57)
Cancer	0.48 (0.17)/0.13 (0.005)	−0.01 (0.15)/−0.004 (0.95)	0.001 (0.15)/ <0.001 (0.99)	−0.004 (0.14)/−0.002 (0.98)
Fear of COVID-19	–	0.20 (0.06)/0.27 (<0.001)	0.14 (0.06)/0.19 (0.01)	0.17 (0.05)/0.26 (<0.001)
Group (Ref: Taiwanese)	1.45 (0.15)/0.60 (<0.001)	−0.75 (0.16)/−0.42 (<0.001)	−1.07 (0.16)/−0.58 (<0.001)	−0.91 (0.14)/−0.56 (<0.001)
**Mode fit statistics**				
*F*-value (*p*-value)	25.70 (<0.001)	6.62 (<0.001)	8.86 (<0.001)	9.38 (<0.001)
*R*^2^ (Adjusted *R*^2^)	0.49 (0.47)	0.21 (0.18)	0.27 (0.24)	0.28 (0.25)

a *Reference group of education is those who had completed secondary school or below*.

*Mouth covering indicates covering mouth when sneezing*.

*Total behavior includes both hand washing and mouth covering when sneezing*.

## Discussion

To the best of the present authors' knowledge, no studies have compared the fear of COVID-19 and preventive COVID-19 infection behaviors between two countries, especially for their older populations. The present study presents important information for healthcare providers and health policy makers, helping them to understand the importance of timing in implementing infection control policies. The fear of COVID-19 was moderate among Iranian older people (scored 3.36 out of a 5-point scale) and low among Taiwanese older people (scored 1.80 of a 5-point scale). The preventive COVID-19 infection behaviors were high in both Iranian older people (scored 4.06–4.15 of a 5-point scale) and Taiwanese older people (scored 4.78–4.86 of a 5-point scale). Moreover, Taiwanese older people as compared with Iranian older people had lower levels of fear of COVID-19 and higher levels of preventive COVID-19 infection behaviors. A significantly positive association was also found between fear of COVID-19 and preventive COVID-19 infection behaviors. Moreover, the association between fear of COVID-19 and preventive COVID-19 infection behaviors was stronger in Iranian older people than in Taiwanese older people.

As compared with the fear found in a general Iranian population (20), the fear of COVID-19 in the present sample was lower. Ahorsu et al. reported a score of approximately 4 from a 5-point scale and the present study reported 3.36 for Iranians and 1.80 for Taiwanese (20). Potential reasons include (i) the communities and governments have better knowledge and information on COVID-19 during the data collection period for the present study; (ii) the governments have applied different methods to correctly disseminate the COVID-19 information for citizens. Indeed, Ahorsu et al. collected data at the peak of COVID-19 infection in Iran (March) and the present study collected the data during a flatter period of COVID-19 infection (April) (20). Some studies (31, 33–35) collected data after Ahorsu et al. also found a lower fear as compared with Ahorsu et al.'s fear findings (20). Therefore, with the governments' efforts in providing correct COVID-19 information, the fear of COVID-19 was not high, even in a higher risk population such as older adults (14, 39).

The effectiveness of disseminating COVID-19 information can be somewhat verified by the high preventive COVID-19 infection behaviors found in the present study. This finding echoes the Protection Motivation Theory (24), the Health Belief Model (22), and the Fear Drive Model (23) that disseminating the potential impact of COVID-19 may improve adherence to performing preventive COVID-19 infection behaviors. More specifically, both Iranian and Taiwanese governments have set up different platforms to disseminate COVID-19 information, including the preventive behaviors (27, 28, 30). Therefore, the extremely high preventive COVID-19 infection behaviors found in the present study may be due to the information dissemination. Another possible explanation for the high preventive COVID-19 infection behaviors is an adequate level of fear of COVID-19. If an individual can properly handle fear, the individual will be aware of the risks of COVID-19 and subsequently take appropriate action to reduce their chances of contractive the virus (21). Indeed, the regression models in the present study found that higher levels of fear were associated with greater preventive behaviors. Moreover, the regression findings justify that the present sample had adequate levels of fear instead of an overwhelming level of fear.

However, an interesting finding is that Taiwanese older people had lower level of fear of COVID-19 but higher levels of preventive COVID-19 behaviors, which contradicts the regression findings on greater fear associated with more preventive COVID-19 behaviors. The main reason may be due to the different levels of COVID-19 severity and government efficiency between the two countries. In Taiwan, the COVID-19 severity was mild and the dissemination of correct COVID-19 preventive behaviors was efficient, which led to low level of fear of COVID-19 and high level of adherence to preventive behaviors. In Iran, the COVID-19 severity was severe and unfortunately the government efficiency in disseminating COVID-19 preventive behaviors was less efficient than Taiwan government (27, 28, 30). Therefore, Iranians as compared with Taiwanese had higher levels of fear and lower levels of adherence to preventive behaviors. However, if we controlled the country effects in the regression models, the results showed that greater fear led to higher levels of adherence to preventive behaviors.

An important finding in the present study is the different levels of fear and preventive behaviors between Iranian and Taiwanese older people. Counter intuitively, Taiwanese older people had less fear but adhered more to preventive behaviors as compared with Iranian older people. A potential explanation is the implementation of infection control policies on Taiwan. With the early reaction in late January (28, 29), the Taiwanese government was able to control the spread of COVID-19 infection and minimize the confirmed cases and deaths. Subsequently, the population, including older people, may feel safe and have more confidence in the government's actions. Regarding the Iranian government, the action taken was slower than the Taiwanese government and the infection rate became hard to control in March (27). Moreover, the COVID-19 outbreak happened to overlap with New Year celebrations in Iran (Persian New Year began on 3 March 2020), which may have made Iranians reluctant to perform preventive COVID-19 infection behaviors as they wanted to celebrate the big event with large family gatherings (27).

There are some limitations in the present study. First, given that different countries have different cultures and habits, the early policies on COVID-19 infection control used in Taiwan may not be applicable to people in Iran. Also, it is unclear whether the effectiveness of such policies is due to the specific populations or due to the early policies that were adopted. Of course, given the nature of this area of research, it is not possible to answer this question directly given experimental designs cannot be employed. A tentative conclusion is that implementing infection control policies in an early stage may be effective in preventing infection spread. Second, there were subtle differences between the two samples (e.g., Iranian older adults were younger than Taiwanese older adults). Thus, the significant differences found in the independent *t*-tests might be due to these subtle differences. The regression models, however, control for these differences. Third, the methods of data collection were different between the Iranian and Taiwanese samples; therefore, it is possible that the different methods used for data collection will cause any answering bias (40). These differences are unlikely to introduce serious bias, as measurement invariant properties between different methods of data collection have been found (41). Fourth, the Iranian participants were recruited using social media and the sample only represents those who were active on the social media and may have sampling bias (42). Fifth, all the measures are based on self-report and at risk of social desirability (e.g., willing to report a high preventive behaviors). Sixth, the representativeness of the present samples is limited because of the use of convenience sampling. Seventh, some important confounders, such as whether participants were contracted with COVID-19, were not assessed. The lack of controlling these confounders may result in biases of our findings. Lastly, given that the present study adopted a cross-sectional design, causality cannot be inferred.

## Conclusion

In conclusion, the present study demonstrated that Iranian older people as compared with Taiwanese older people had higher levels of fear of COVID-19 but implemented a lower frequency of preventive COVID-19 infection behaviors. Such findings remained even when controlling for important confounders (i.e., age, gender, educational level, living area, and various chronic diseases). Moreover, higher levels of fear of COVID-19 were associated with more preventive COVID-19 infection behaviors. However, the level of fear of COVID-19 in the present study was not high (3.36 out of 5 from Iranians and 1.80 out of 5 from Taiwanese). The lower level of fear, but higher level of preventative behaviors in the Taiwanese sample, may reflect the benefits of their government's early and swift reaction to the COVID-19 infection.

## Data Availability Statement

The raw data supporting the conclusions of this article will be made available by the authors, without undue reservation.

## Ethics Statement

The studies involving human participants were reviewed and approved by Taipei Medical University's ethical committee approved the study with registered numbers of TMU-JIRB N202005044. Also, ethics committee of Qazvin University of Medical Sciences approved the study with registered number of IR.QUMS.REC.1398.375. The patients/participants provided their written informed consent to participate in this study.

## Author Contributions

AP, C-hL, W-LH, Y-PC, Y-JK, Y-PL, C-YL, and DS contributed conception and design of the study. C-hL, W-LH, Y-PC, and Y-JK organized the database. AP and C-YL performed the statistical analysis and wrote the first draft of the manuscript. AP, C-hL, W-LH, Y-PL, and C-YL interpreted the results. C-hL, Y-PC, and Y-JK wrote sections of the manuscript. C-hL, W-LH, Y-PL, C-YL, and DS critically review the manuscript. All authors contributed to manuscript revision, read, and approved the submitted version.

## Funding

This study was supported in part by a research grant from the Ministry of Science and Technology, Taiwan (MOST109-2327-B-006-005) and in part by a research grant from the Taipei Municipal Wanfang Hospital Cross-Institutions Fund (110-swf-01).

## Conflict of Interest

The authors declare that the research was conducted in the absence of any commercial or financial relationships that could be construed as a potential conflict of interest.

## Publisher's Note

All claims expressed in this article are solely those of the authors and do not necessarily represent those of their affiliated organizations, or those of the publisher, the editors and the reviewers. Any product that may be evaluated in this article, or claim that may be made by its manufacturer, is not guaranteed or endorsed by the publisher.
